# Dysphagia Screening in an Aging Population: A Scoping Review of Structural Gaps Between Triage and Comprehensive Evaluation in International and Japanese Practices

**DOI:** 10.1002/jgf2.70150

**Published:** 2026-07-14

**Authors:** Akihito Ueda, Kanji Nohara

**Affiliations:** ^1^ Medical Corporation Toujinkai, Fujitate Hospital Osaka Japan; ^2^ Faculty of Pharmaceutical Sciences Teikyo Heisei University Tokyo Japan; ^3^ Department of Rehabilitation for Orofacial Disorders Osaka University Graduate School of Dentistry Osaka Japan

**Keywords:** aging population, dysphagia screening, post‐screening management, referral pathway, scoping review

## Abstract

This scoping review aimed to map the characteristics of dysphagia screening implementation in published studies and to compare operational differences between non‐Japanese and Japanese practices, focusing on screening personnel, screening tools, and post‐screening management pathways. Following the Joanna Briggs Institute methodology and PRISMA‐ScR guidelines, PubMed, Cochrane Library, CINAHL, and Ichushi‐Web were searched for studies published between 2016 and 2025 that described the clinical implementation of dysphagia screening. Twenty‐nine studies from 12 countries were included. In all 21 non‐Japanese studies, nurses performed screening using a single validated tool, with referral to speech‐language therapists for comprehensive evaluation. Japanese studies (*n* = 8) demonstrated variability, with screening conducted by nurses (*n* = 3), speech‐language therapists (*n* = 3), and physicians (*n* = 2), who frequently use multiple test combinations. Only 2 of 8 Japanese studies documented referral pathways for comprehensive evaluation following positive screening. Non‐Japanese studies consistently implemented structured screening‐to‐evaluation pathways, whereas Japanese studies demonstrated variability in screening personnel and limited documentation of post‐screening referral processes, resulting in blurring of the conceptual distinction between triage and diagnostic evaluation. As Japan's broader screening approach extends beyond the high‐risk populations targeted internationally, establishing standardized post‐screening pathways with clear role delineation becomes essential to maintain screening quality and optimize resource allocation in aging populations.

## 1 Introduction

Oropharyngeal dysphagia is highly prevalent among older adults, affecting approximately 15%–30% of community‐dwelling individuals and more than 50% of nursing home residents [1, 2]. When unrecognized or untreated, dysphagia can result in malnutrition, dehydration, and aspiration pneumonia [3]. These complications contribute to increased morbidity, prolonged hospitalization, and higher mortality in this population [4]. Aspiration pneumonia represents a leading cause of death among older adults, and patients with dysphagia have a threefold higher risk of developing pneumonia than those without swallowing impairment [5]. Consequently, systematic dysphagia screening has been increasingly recognized as a critical component of patient safety and quality care across healthcare settings [6].

In Western countries, clinical practice guidelines provide explicit recommendations for dysphagia screening. The American Heart Association (AHA) and the American Stroke Association (ASA) published a state‐of‐the‐art conference proceeding in 2013 recommending that patients with acute stroke undergo dysphagia screening before initiation of oral intake. Screening should be conducted by trained healthcare professionals, most commonly nurses, with referral to speech‐language therapists (STs; equivalent to speech‐language pathologists in North America and Australia) for comprehensive assessment if patients fail the initial screening [7]. Similarly, the Scottish Intercollegiate Guidelines Network recommends early dysphagia screening using validated bedside assessments, typically water swallow tests administered by trained nursing staff, followed by prompt referral to STs when screening indicates risk of aspiration [8]. These guidelines define screening procedures, delineate professional roles, and establish standardized management pathways for patients identified as at risk.

In Japan, where population aging is associated with high rates of dysphagia and aspiration pneumonia [9, 10], the Japanese Society of Dysphagia Rehabilitation published clinical practice guidelines in 2019 recommending dysphagia screening to facilitate early identification of suspected swallowing disorders and timely referral for further evaluation and treatment [11]. However, unlike Western guidelines, the Japanese recommendations do not clearly specify which healthcare professionals should perform screening or provide detailed protocols for managing patients who fail initial screening. This limited operational guidance may contribute to variability in screening practices across Japanese healthcare institutions.

Despite widespread acknowledgment of the importance of dysphagia screening in clinical guidelines, to our knowledge, no systematic review has comprehensively mapped how screening is implemented across healthcare settings and countries. Critical operational components, including the professionals responsible for screening, the tools and protocols used, and the management strategies for patients who fail screening, remain inconsistently reported in the literature. Clarifying these implementation characteristics is essential for identifying best practices, addressing gaps in current approaches, and informing evidence‐based recommendations to optimize dysphagia screening globally.

Dysphagia screening has direct implications for general medical practice. Aspiration pneumonia is one of the most common reasons for hospitalization among older adults. According to the British Thoracic Society Clinical Statement, this condition accounts for more than 60% of community‐acquired pneumonia hospitalizations in Japan, compared with only 5%–15% in Western countries [12]. Komiya et al. [13] previously reported that approximately 73% of older patients with pneumonia were treated by non‐pulmonologists, such as general internists and primary care physicians, highlighting the central role that generalist physicians play in the frontline management of patients with aspiration pneumonia and related conditions. In Japan, where the aging population necessitates screening beyond narrowly defined high‐risk groups, the absence of standardized post‐screening pathways may mean that clinical decision making regarding oral intake and diet modification proceeds without distinct comprehensive evaluation. This situation is particularly relevant to general practitioners who routinely manage hospitalized older adults and must navigate the boundary between initial screening and the need for specialist referral. Understanding how different healthcare systems operationalize the transition from screening to comprehensive evaluation is therefore essential for optimizing dysphagia management in aging populations.

Accordingly, this scoping review aimed to systematically identify and map the characteristics of dysphagia screening implementation reported in published studies, with particular emphasis on differences between Japanese and non‐Japanese practice contexts. Specifically, we examined [1] which healthcare professionals perform dysphagia screening in various clinical settings, [2] which screening tools and protocols are used, and [3] how patients who fail screening are managed across geographical and clinical contexts.

## 2 Materials and Methods

### 2.1 Protocol and Registration

The protocol for this scoping review was prospectively registered with the Open Science Framework (https://osf.io/ahbqs/). The review was conducted in accordance with the Joanna Briggs Institute (JBI) methodology for scoping reviews [14] and is reported following the Preferred Reporting Items for Systematic Reviews and Meta‐Analyses Extension for Scoping Reviews (PRISMA‐ScR) [15].

### 2.2 Objective

In this scoping review, we aimed to identify and map the global characteristics of dysphagia screening implementation, with particular emphasis on operational differences between studies conducted in Japan and those conducted elsewhere. Studies were classified as “Japanese” if conducted in Japan, irrespective of publication language, and as “non‐Japanese” if conducted in any other country. This classification reflected the primary research objective: to characterize Japanese practice in relation to international practice, rather than to compare specific geographic regions.

### 2.3 Eligibility Criteria

The eligibility criteria were defined using the Population, Concept, Context (PCC) framework, as follows: Population: Adult patients (≥ 18 years) in any clinical setting (studies exclusively involving pediatric populations were excluded). Concept: We included studies that reported on the clinical implementation, workflow, or operational protocols of dysphagia screening, specifically those describing “who” performs the screening, “how” decisions are made (e.g., NPO vs. diet modification), and the organizational outcomes of implementation. To distinguish “implementation research” from “instrument validation research,” we explicitly excluded psychometric validation studies, diagnostic accuracy studies without implementation details, and risk factor analyses that did not involve a screening protocol. Context: All healthcare settings were eligible, including acute care hospitals, rehabilitation facilities, and long‐term care institutions, to capture international variation in practice.

### 2.4 Information Sources and Search Strategy

Four electronic databases were searched: PubMed, Cochrane Library, CINAHL, and Ichushi‐Web (Japan Medical Abstracts Society). The search was conducted on January 3, 2026, and limited to studies published between January 1, 2016, and December 31, 2025, to capture contemporary dysphagia screening practices over the preceding decade. During this interval, the Japanese Society of Dysphagia Rehabilitation issued updated assessment guidelines [11], which informed current clinical practice.

In PubMed, Cochrane Library, and CINAHL, the following search strategy was applied: (dysphagia OR “swallowing disorder*”) AND (screening OR “bedside assessment” OR “swallow* test”) AND (nurse* OR “speech therapist*” OR “speech‐language therapist*” OR “speech‐language pathologist*” OR “occupational therapist*” OR dentist* OR “dental hygienist*” OR physician* OR “healthcare professional*” OR physiotherapist* OR “physical therapist*” OR “care worker*” OR “healthcare assistant*”). Results were restricted to human studies published in English. In Ichushi‐Web, equivalent Japanese‐language terms for dysphagia, screening, and healthcare professionals were used. Filters were applied to exclude case reports and conference proceedings.

### 2.5 Study Selection

All retrieved citations were imported into the Rayyan software [16], and duplicates were removed. One reviewer performed the initial title and abstract screening, and a second reviewer independently verified eligibility. Articles meeting the inclusion criteria underwent full‐text review. Two reviewers resolved discrepancies through discussion and consensus. Reasons for exclusion at the full‐text stage were systematically documented and categorized according to the PCC framework to clarify gaps in the literature.

### 2.6 Data Charting

One reviewer extracted data using a standardized data‐charting form, and a second reviewer verified accuracy and completeness. Extracted variables included author, publication year, country, clinical setting, target population, screening tool characteristics, assessor qualifications and training requirements, and management protocols for failed screening results. Disagreements were resolved through discussion between the reviewers.

## 3 Results

### 3.1 Selection of Sources

Database searches identified 1645 records (PubMed: 1244; Cochrane Library: 73 [reviews: 1, trials: 72]; CINAHL: 155; Ichushi‐Web: 173). After removal of 118 duplicates, 1527 records underwent title and abstract screening, and 1402 were excluded as not relevant to the study objective.

A total of 125 full‐text articles were assessed for eligibility. Ninety‐six articles were excluded for the following reasons: PCC mismatch (*n* = 75), including validation or development studies (*n* = 32), risk factor or prognostic analyses (*n* = 22), insufficient implementation detail (*n* = 18), and prevalence studies (*n* = 3); surveys or quality‐of‐life studies (*n* = 13); reviews or guidelines (*n* = 4); irrelevant methodologies such as artificial intelligence–based models or treatment‐only studies (*n* = 3); and diagnostic assessments without screening components (*n* = 1).

**FIGURE 1:**
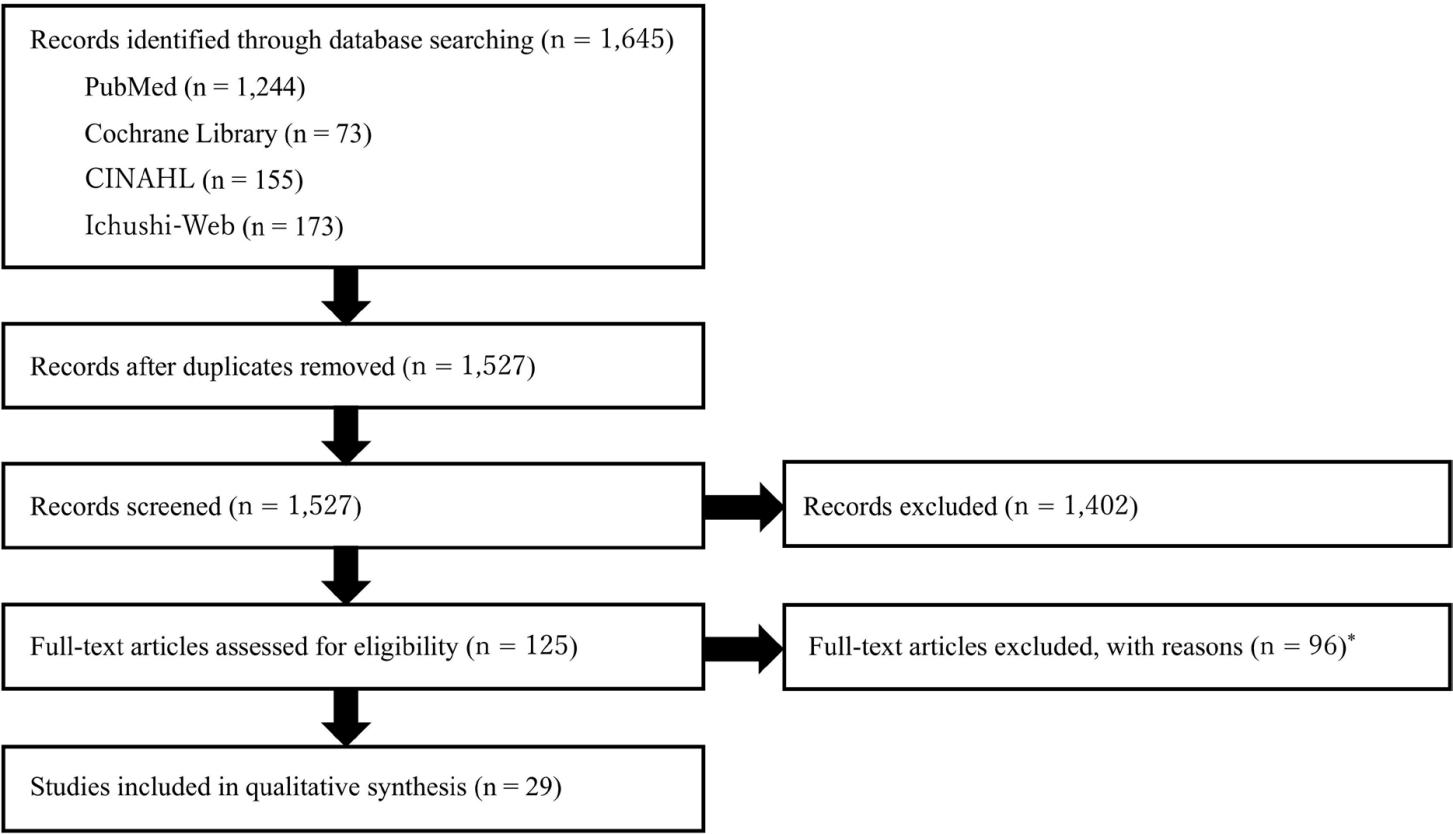
PRISMA flow diagram of study selection. *Reasons for exclusion: PCC mismatch (*n* = 75), surveys/QOL studies (*n* = 13), reviews/guidelines (*n* = 4), irrelevant methodology (*n* = 3), diagnostic assessment only (*n* = 1).

Ultimately, 29 studies met the inclusion criteria and were included in the review (Figure 1).

### 3.2 Characteristics of Included Studies

Twenty‐nine studies met the inclusion criteria (Table 1). These studies were conducted in 12 countries. Among the 24 English‐language publications [6, 17, 18, 19, 20, 21, 22, 23, 24, 25, 26, 27, 28, 29, 30, 31, 32, 33, 34, 35, 36, 37, 38, 44], Australia contributed the largest number of studies (*n* = 6), followed by China (*n* = 3), Japan (*n* = 3), Canada (*n* = 2), and the United States (*n* = 2). Single studies originated from New Zealand, the Netherlands, Singapore, Switzerland/Finland, Austria, Portugal, and Brazil. One multinational study included 17 European countries. In addition, five Japanese‐language studies were identified from Japan [39, 40, 41, 42, 43].

**TABLE 1:** Characteristics of included studies.

ID	Study	Country	Setting	Target population	Screener	Tool	Management of failed screening
Group A: English‐language publications (*n* = 24)							
A‐1	Liu et al. [17]	China	Neurology (Stroke)	Acute stroke patients	Nurse	SSA	Referral to ST
A‐2	Middleton et al. [18]	Australia	Stroke services	Acute stroke patients	Nurse	ASSIST	Referral to ST
A‐3	See et al. [19]	Singapore	Medical ICU	Critically ill medical patients (Post‐extubation)	Nurse	Modified Massey Bedside Swallowing Screen	Referral to ST
A‐4	Palli et al. [6]	Austria	Neurology (Stroke)	Acute stroke patients	Nurse	GUSS	Referral to ST
A‐5	Schefold et al. [20]	Switzerland/Finland	ICU	Mechanically ventilated ICU patients	Nurse	WST	Referral to ST
A‐6	Christensen and Trapl [21]	Australia	ICU	Post‐extubation patients	Nurse	GUSS‐ICU	Referral to ST
A‐7	O'Malley et al. [22]	USA	General medicine	General medicine inpatients	Nurse	SITUP Protocol	Referral to ST
A‐8	Miki et al. [23]	Japan	Gastric surgery	Elderly patients with gastric cancer	Physician/Team	Symptom Q + MWST + RSST	Multidisciplinary team conference
A‐9	Omura et al. [24]	Japan	Mixed ICU	Post‐extubation patients	Nurse	MWST + WST + Food Test	Referral to ST/Team
A‐10	Middleton et al. [25]	Australia	Acute stroke unit	Acute stroke patients	Nurse	ASSIST	Referral to ST
A‐11	Heaton et al. [26]	Australia	Hospital‐wide	All adult inpatients (Hospital‐wide)	Nurse	RBWH Dysphagia Screening Tool	Referral to ST
A‐12	Murray et al. [27]	Australia	Stroke unit	Acute stroke patients	Nurse	Adelaide Swallow Screen	Referral to ST
A‐13	Taveira et al. [28]	Portugal	Internal medicine	Internal medicine patients	Nurse	V‐VST	Referral to ST
A‐14	Wijnen et al. [29]	Netherlands	Orthogeriatric ward	Patients with hip fracture	Nurse	SSA	Referral to ST
A‐15	Liu et al. [30]	China	Neurology (Stroke)	Acute stroke patients	Nurse	EAT‐10 + Water Swallow Test	Referral to ST
A‐16	Miles et al. [31]	New Zealand	CVICU	Post‐cardiothoracic surgery patients	Nurse	NDS	Referral to ST
A‐17	Barker et al. [32]	Canada	ICU	Post‐extubation patients	Nurse	SAPE	Referral to ST
A‐18	Jones et al. [33]	Australia	Emergency department/Stroke	Acute stroke patients (ED)	Nurse	FeSS Protocols (ASSIST)	Referral to ST
A‐19	Kanzawa et al. [34]	Japan	General Medicine	Patients with aspiration pneumonia	Physician (Non‐expert)	Dysphagia Assessment Protocol	Physician decision (Protocol)
A‐20	Middleton et al. [25]	Europe (17 countries)	Stroke Units	Acute stroke patients	Nurse	FeSS Protocols	Referral to ST
A‐21	Shen et al. [35]	China	Neurology (Stroke)	Acute stroke patients	Nurse	WST + SpO_2_ Monitoring	Referral to ST
A‐22	Wedemire et al. [36]	Canada	ICU	ICU patients	Nurse	Yale Swallow Protocol	Referral to ST
A‐23	Garcia et al. [37]	Brazil	Adult ICU	Post‐extubation patients	Nurse	Customized GUSS	Referral to ST
A‐24	Koirala et al. [38]	USA	Neurological ICU	Acute stroke patients	Nurse	Yale Swallow Protocol	Referral to ST
Group B: Japanese‐language publications (*n* = 5)							
B‐1	Yamazaki and Fujimori [39]	Japan	Acute Care (Hospital‐wide)	High‐risk inpatients (Admission)	Nurse	Modified Aspiration Risk Screening	Ward nurse management/Team
B‐2	Kitamura et al. [40]	Japan	University Hospital (All wards)	All admitted patients	Nurse	EAT‐10 + MWST	Referral to ST/Team
B‐3	Asakuma et al. [41]	Japan	Orthopedics	Patients with proximal femoral fracture	ST	ST Dysphagia Risk Protocol	ST assessment (Direct)
B‐4	Osanai et al. [42]	Japan	Acute Care	Patients with aspiration pneumonia	ST	Aspiration Pneumonia Assessment Criteria	Early ST rehabilitation (Direct)
B‐5	Tatebe et al. [43]	Japan	General Medicine	Patients with aspiration pneumonia	ST/Multidisciplinary	Dysphagia Assessment Flow	Multidisciplinary team conference

Abbreviations: ASSIST, Acute Screening of Swallowing in Stroke/TIA; CVICU, Cardiovascular Intensive Care Unit; EAT‐10, Eating Assessment Tool‐10; FeSS, Fever, Sugar, Swallow; GUSS, Gugging Swallowing Screen; ICU, Intensive Care Unit; MDT, Multidisciplinary Team; MWST, Modified Water Swallow Test; NDS, Nurse Dysphagia Screen; RSST, Repetitive Saliva Swallowing Test; SAPE, Swallowing Algorithm Post‐Extubation; SSA, Standardized Swallowing Assessment; ST, Speech‐Language Therapist (equivalent to Speech‐Language Pathologist in North America and Australia); V‐VST, Volume‐Viscosity Swallow Test; WST, Water Swallow Test.

Stroke units or neurology wards represented the most common clinical setting (*n* = 11), followed by intensive care units (*n* = 8). General medical wards accounted for four studies, three studies implemented hospital‐wide screening programs, and three were conducted in orthopedic or surgical settings.

### 3.3 Screening Personnel

Nurses were the primary screening personnel in most studies (*n* = 24). Speech therapists (STs) or ST‐led multidisciplinary teams conducted screening in three studies, and physicians or physician‐led teams in two studies. In all 21 non‐Japanese studies, nurses performed screening. In contrast, the eight Japanese studies demonstrated greater variability: screening was conducted by nurses (*n* = 3), STs or ST‐led teams (*n* = 3), and physicians or physician‐led teams (*n* = 2).

### 3.4 Screening Tools and Protocols

A range of screening tools was reported. Non‐Japanese studies predominantly employed a single validated instrument, including the Yale Swallow Protocol, the Gugging Swallowing Screen (GUSS), standardized water swallow tests, and the Acute Screening of Swallow in Stroke or TIA (ASSIST) protocol and its derivatives, which were used in four stroke‐focused studies from Australia and Europe. Japanese studies more frequently implemented combinations of multiple assessments, such as the Modified Water Swallow Test (MWST), the Repetitive Saliva Swallowing Test (RSST), and food‐based swallowing tests.

### 3.5 Management of Patients Who Failed Screening

In all 21 non‐Japanese studies, patients who failed screening were referred for comprehensive swallowing evaluation. In contrast, the eight Japanese studies demonstrated substantial heterogeneity in post‐screening management: referral to STs with multidisciplinary team involvement (*n* = 2), multidisciplinary case conferences (*n* = 2), direct ST management without a clearly documented referral pathway (*n* = 2), physician‐led protocol‐based decision‐making (*n* = 1), and ward‐level management with team support (*n* = 1). Only two of the eight Japanese studies explicitly documented a referral pathway for comprehensive evaluation following positive screening, compared with all non‐Japanese studies. Key differences between non‐Japanese and Japanese studies are summarized in Table 2.

**TABLE 2:** Comparison of dysphagia screening implementation: Non‐Japanese vs. Japanese studies.

Feature	Non‐Japanese studies (*n* = 21)	Japanese studies (*n* = 8)
Number of studies	21	8 (3 English, 5 Japanese)
Clinical settings	Predominantly stroke units (*n* = 10) and ICUs (*n* = 7); also general medicine (*n* = 2), hospital‐wide (*n* = 1), orthopedics (*n* = 1)	Broader range: general medicine (*n* = 2), gastric surgery (*n* = 1), orthopedics (*n* = 1), ICU (*n* = 1), hospital‐wide (*n* = 2), acute care (*n* = 1)
Target population	Condition‐specific: acute stroke patients, post‐extubation patients, post‐surgical patients	Broader: includes general elderly inpatients, all admitted patients, and aspiration pneumonia patients
Primary screener	Nurses in all 21 studies (100%)	Nurses (*n* = 3, 37.5%), STs or ST‐led teams (*n* = 3, 37.5%), physicians or physician‐led teams (*n* = 2, 25%)
Screening tools	Single validated tool per study (e.g., GUSS, Yale Swallow Protocol, ASSIST, SSA, V‐VST)	Frequently combinations of multiple tests (e.g., MWST + RSST + Food Test, EAT‐10 + MWST, Symptom Q + MWST + RSST)
Management of failed screening	Consistent: referral to ST for comprehensive evaluation (21/21)	Heterogeneous: ST referral with team involvement (*n* = 2) MDT conference (*n* = 2) Direct ST management without referral pathway (*n* = 2) Physician‐led protocol decision (*n* = 1) Ward‐level management with team support (*n* = 1)
Screening–evaluation distinction	Clear two‐stage process: screening by nurses → referral to ST for comprehensive evaluation	Less clearly delineated: screening and evaluation roles overlap; referral pathways for comprehensive evaluation were documented in only 2 of 8 studies

Abbreviations: ASSIST, Acute Screening of Swallowing in Stroke/TIA; EAT‐10, Eating Assessment Tool‐10; GUSS, Gugging Swallowing Screen; ICU, Intensive Care Unit; MDT, Multidisciplinary Team; MWST, Modified Water Swallow Test; RSST, Repetitive Saliva Swallowing Test; SSA, Standardized Swallowing Assessment; ST, Speech‐Language Therapist; V‐VST, Volume‐Viscosity Swallow Test.

## 4 Discussion

This scoping review identified 29 studies examining the implementation of dysphagia screening across 12 countries. The findings demonstrated substantive differences between Japanese and non‐Japanese studies in three principal domains: the professional responsible for screening, the clinical contexts in which screening is initiated, and the management pathways for patients who fail screening.

Non‐Japanese studies primarily focused on well‐defined clinical contexts in which the risk of new‐onset dysphagia is established. The AHA/ASA guidelines recommend dysphagia screening for patients with acute stroke [45], and professional society guidelines similarly endorse screening for patients at high risk of post‐extubation dysphagia in intensive care settings [46]. In contrast, Japanese studies reported screening across broader populations, including general older inpatients and community‐dwelling older adults. This broader approach likely reflects Japan's demographic context as the world's most aged society, where the prevalence of dysphagia [9] and aspiration pneumonia [10] exceeds that reported in many other countries. Accordingly, Japan's population‐based screening strategy may represent a pragmatic response to advanced population aging rather than a limitation in clinical rigor. However, Japanese guidelines do not clearly specify professional accountability or explicit clinical triggers for initiating screening. This lack of specification may contribute to variability in implementation across health care settings. Although the broader approach is contextually appropriate, clearer specification of responsibility and initiation criteria would improve standardization and reproducibility.

Screening is defined as a triage process designed to identify individuals who require further diagnostic evaluation, whereas diagnostic evaluation aims to confirm the presence of a disorder and characterize its underlying mechanisms. In Western health care systems, this distinction is generally operationalized through a structured two‐stage pathway: nurses conduct initial screening, and patients who fail screening are referred to STs, who typically hold autonomous authority to perform instrumental diagnostic assessments, including videofluoroscopy and fiberoptic endoscopic evaluation of swallowing. In contrast, this separation appears less clearly delineated in Japanese practice. The review identified variability in the professionals responsible for screening, including nurses, STs, and physicians, and Japanese STs generally do not possess the same degree of autonomous diagnostic authority as their Western counterparts.

This finding has direct implications for general medical practice. In many clinical settings, generalist physicians are responsible for making decisions regarding oral intake resumption and diet texture after screening, particularly when specialist evaluation is not immediately available. When post‐screening referral pathways are not clearly defined, generalists may be required to make clinical decisions based on screening results alone, without the benefit of any comprehensive diagnostic evaluation. This raises concerns regarding both premature resumption of oral feeding, which increases aspiration risk, and unnecessarily prolonged fasting, which may compromise nutritional status and delay recovery.

The blurring of professional roles has important implications for both resource allocation and the organization of patient care pathways. When specialists conduct initial screening, health care resources may be deployed inefficiently because screening is intended to be performed by trained frontline staff. More critically, the post‐screening clinical pathway appears to differ across regions. In Japanese studies, care pathways frequently demonstrated direct progression to multidisciplinary team conferences or therapeutic intervention without documentation of a comprehensive diagnostic evaluation [23, 43]. This pattern raises concern that screening outcomes may be used to inform clinical decision‐making in the absence of a distinct confirmatory evaluation step.

In non‐Japanese studies, this risk appears to be mitigated by clearer delineation of professional responsibilities: nurses perform initial screening, and patients who fail screening are referred for comprehensive diagnostic evaluation. This structured model preserves the function of screening as a triage mechanism, while diagnostic decisions are made by professionals with appropriate expertise. The consistent adoption of this two‐stage approach across the 21 non‐Japanese studies suggests that formal separation between the screening and evaluating professionals may help maintain the conceptual and operational distinction between screening and diagnosis.

This review has several strengths. It represents the first systematic mapping of dysphagia screening implementation comparing Japanese and non‐Japanese practices. In contrast to validation studies that focus primarily on test accuracy, this review examined operational implementation and therefore captured the real‐world context in which screening is conducted. Inclusion of both English‐ and Japanese‐language literature enabled more comprehensive representation of Japanese practice, which would likely be underrepresented in English‐only reviews. Application of the Population–Concept–Context (PCC) framework with explicit inclusion criteria further distinguished implementation research from instrument validation studies.

This review also has important limitations. As a scoping review, we did not appraise the methodological quality of included studies or perform a quantitative synthesis of outcomes. The search was restricted to English‐ and Japanese‐language publications, potentially excluding relevant studies published in other languages. Unpublished literature was not included, which may have introduced publication bias. Substantial heterogeneity in study design, clinical setting, and outcome measurement precluded quantitative comparison of screening effectiveness. The search was limited to studies published between 2016 and 2025 and may therefore have excluded earlier implementation models. Finally, cross‐national differences in the scope of practice of STs represent structural variations that influence how screening systems can be organized and interpreted.

These findings have implications for the organization of dysphagia screening in clinical practice. The structured two‐stage approach consistently observed in non‐Japanese studies (initial screening by trained healthcare professionals followed by referral for comprehensive evaluation) ensures that clinical decisions are informed by diagnostic‐level assessment. When screening and evaluation roles are structurally separated, referral for comprehensive evaluation becomes a distinct subsequent stage, preserving the conceptual distinction between screening and diagnosis. This separation may reduce the blurring of screening and diagnostic evaluation observed in Japanese practice.

The Japan Academy of Nursing Science has published clinical practice guidelines recommending that nurses assess aspiration risk and pharyngeal residue using bedside screening tools, including the RSST, MWST, and food tests [47]. However, the findings of this review indicate that these recommendations have not been widely translated into routine clinical screening practice, as only three of eight Japanese studies identified nurses as primary screeners. More critically, the establishment of a structured post‐screening evaluation framework is essential regardless of which professionals perform screening. This framework should define referral criteria for patients who fail screening and designate the professionals or multidisciplinary teams responsible for comprehensive evaluation at each institution. The variability observed across Japanese studies may partly reflect differences in the availability of established evaluation infrastructures across clinical settings.

These considerations are particularly relevant in the context of Japan's super‐aged society, where dysphagia screening must be applied to broader populations than solely condition‐specific groups (acute stroke, post‐extubation) currently targeted by international guidelines. The expansion of screening indications without the concurrent establishment of post‐screening infrastructure creates an operational gap: more patients are identified as potentially at risk; however, the systems for managing positive screening results remain fragmented. For generalist physicians managing older inpatients, the availability of a clearly defined post‐screening evaluation pathway determines whether screening can function as an effective triage mechanism, or merely generates clinical uncertainty regarding oral intake management.

In addition, guidelines could more clearly specify the clinical situations that trigger screening. Non‐Japanese studies focused on well‐defined high‐risk populations, such as patients with acute stroke or those at risk of post‐extubation dysphagia, whereas Japanese studies applied screening to broader populations, including general older inpatients. Although broader screening may be justified given Japan's demographic context, explicit criteria for screening indications could reduce inter‐institutional variability. Future research should aim to evaluate the feasibility and clinical outcomes of structured dysphagia screening systems in Japanese health care settings, including the establishment of standardized post‐screening evaluation pathways tailored to different clinical contexts.

## 5 Conclusion

In conclusion, this scoping review demonstrated that non‐Japanese studies consistently employed structured screening‐to‐evaluation pathways with clear role delineation, whereas Japanese studies exhibited variability in screening personnel and less clearly defined post‐screening management processes. The limited documentation of referral pathways for comprehensive evaluation in Japanese studies represents a structural concern that warrants attention from clinicians and policymakers. As aging populations worldwide increasingly require dysphagia screening beyond the currently narrowly defined high‐risk groups, establishing standardized post‐screening evaluation frameworks with clear role delineation will be essential. These findings will be particularly relevant for generalist physicians who routinely manage older inpatients and must determine whether screening‐positive patients require specialist referral for comprehensive evaluation or can be safely managed based on screening results alone.

The completed PRISMA‐ScR checklist is provided as Appendix S1.

## Author Contributions

**Akihito Ueda:** conceptualization, methodology, data curation, investigation, project administration, writing – original draft, writing – review and editing. **Kanji Nohara:** methodology, investigation, data curation, writing – review and editing, validation.

## Funding

The authors have nothing to report.

## Ethics Statement

The authors have nothing to report.

## Consent

The authors have nothing to report.

## Conflicts of Interest

The authors declare no conflicts of interest.

## Supporting information


**Appendix S1:** Preferred Reporting Items for Systematic reviews and Meta‐Analyses extension for Scoping Reviews (PRISMA‐ScR) Checklist.

## Data Availability

All data generated and/or analyzed during this study are included in this published article and its tables.
